# Short-term exposure to various ambient air pollutants and emergency department visits for cause-stable ischemic heart disease: a time-series study in Shanghai, China

**DOI:** 10.1038/s41598-023-44321-1

**Published:** 2023-10-09

**Authors:** Yonghong Zhou, Yi Jin, Zheng Zhang

**Affiliations:** 1 Affiliated Renhe Hospital of Shanghai University (Renhe Hospital, Baoshan District), School of Medicine, Shanghai University, Shanghai, China; 2 Department of Radiation Oncology, Hunan Cancer Hospital, The Affiliated Cancer Hospital of Xiangya School of Medicine, Central South University, Changsha, Hunan China; 3 Service of Endocrinology, Affiliated Renhe Hospital of Shanghai University (Renhe Hospital, Baoshan District), Shanghai, China

**Keywords:** Environmental social sciences, Cardiology, Risk factors

## Abstract

Studying the impact of local meteorological conditions and air pollution on cardiovascular disease is crucial for reducing the burden of cardiovascular disease. However, there have been few studies on the acute effects of various air pollutants on stable ischemic heart disease (SIHD), and the effects of these factors are not well defined and require further investigation. We performed a time-series study aimed at exploring the association between short-term exposure to various air pollutants and emergency department (ED) visits for SIHD during 2013–2020 in Baoshan District Renhe Hospital of Shanghai, China. The associations between air pollution (NO_2_, PM_2.5_, PM_10_, SO_2_ O_3_-8 h and CO) and ED visits were analyzed using quasi-Poisson regression. Subgroup and sensitivity analyses were conducted. From 2013 to 2020, a total of 18,241 ED visits for SIHD were recorded. Elevated PM_2.5_, PM_10_, NO_2_, SO_2_ and CO were significantly associated with increased ED visits for SIHD at lag (0, 5), lag 0, lag (0-4, 01-03), lag (0-3, 5, 01-03) and lag (3-5). When the concentration of O_3_-8 h was lower than the threshold recommended by the WHO, exposure to O_3_-8 h was associated with a slightly decreased risk of SIHD. Moreover, the relationship between different types of air pollution and the frequency of ED visits exhibited variations based on gender, age, and seasonality. This study suggests that short-term exposure to PM_2.5_, PM_10_, NO_2_, SO_2_ and CO might induce SIHD, especially in old females. Air pollution control measures should be encouraged to prevent the occurrence and development of SIHD.

## Introduction

Coronary heart disease (CHD) also called ischemic heart disease (IHD), one of the most serious cardiovascular disease^1^, contributing significantly to global mortality and morbidity rates^2^. Stable ischemic heart disease (SIHD) is a syndrome marked by stable angina, which refers to pain or pressure brought on reliably by exertion or emotion and relieved by rest or nitroglycerin^3^. This may or may not be associated with obstructive IDH^3^. SIHD is a major contributor to mortality rates in the United States and numerous other countries^4^. Therefore, identifying risk factors for SIHD is crucial to develop more efficient strategies for its management and prevention.

It is widely accepted that environmental factors play an important role in the occurrence and development of cardiovascular diseases. Exposure to adverse atmospheric conditions and various air pollutants is associated with an increased risk of CHD and mortality^5^. Both low and high air temperatures have been identified as risk factors for cardiovascular diseases^6,7^ and have been associated with increased mortality rates^8^.

In addition, there is mounting evidence that certain ambient air pollutants are linked to an elevated risk of CHD. A systematic review of 26 studies investigated the adverse effects of short-term exposure to air pollution associated with CHD and found that such exposure can increase the incidence of adverse cardiovascular events and the risk of death from cardiovascular causes^9^. Short-term exposure to PM_2.5_ has been reported to be associated with a 1~3% increase in the risk of acute cardiovascular events^10^, and PM_10_ exposure is also positively associated with the number of hospitalizations for CHD^11,12^. Apart from particulate pollution, short-term exposure to NO_2_ was also found to be positively associated with the number of hospitalizations for CHD^11^.

With the intensification of global climate change and industrialization, meteorological conditions and air pollution have become one of the main factors leading to the occurrence and exacerbation of cardiovascular disease. The incidence of CHD continues to rise every year in China^13^. Moreover, according to Shanghai Health Statistics Reports in 2021^14^, the specific mortality rate of the cardiovascular system in Shanghai was 391.50 per 10^5, making it the leading cause of death. It is important for individuals with SIHD to take extra precautions when exposed to multiple air pollutants. Air pollution control efforts should adopt a comprehensive strategy that considers the simultaneous effects of various pollutants. In addition, implementing public health and environmental measures to reduce air pollution can help mitigate the global trend of increasing cardiovascular disease. However, few previous studies have explored the relationships between various types of air pollution and ED visits for SIHD^15–20^. Moreover, no research has been conducted to simultaneously investigate the relationships between six different types of air pollutants (PM_2.5_, PM_10_, NO_2_, SO_2_, CO, and O_3_-8h) and SIHD. Therefore, our study aimed to investigate the correlation between short-term exposure to different types of air pollution and ED visits for SIHD in Shanghai, China, from 2013 to 2020.

## Methods

### Study settings

This study was conducted in Shanghai, China (Fig. S1), which is located at a latitude of 31.12° N and a longitude of 121.30° E. Shanghai experiences a subtropical monsoon climate, with hot and rainy summers and cold and relatively dry winters^21^. Shanghai, with a population of 24.8 million in 2022, boasts the largest urban area in China. It serves as a prominent international hub for economic, financial, trade, shipping, and innovation activities within the country^22^.

### Emergency department (ED) visit data

ED visit records were extracted from the computerized medical database of Renhe Hospital in Baoshan District, Shanghai, China, from January 1, 2013, to December 31, 2020. This hospital is a comprehensive hospital in Shanghai equipped with 360 beds and catering to over 0.7 million outpatients each year. Detailed patient information, such as visit dates, age, gender, and clinical diagnoses, was collected for analysis. The diagnoses of all patients in our study according to the tenth edition of the International Classification of Disease (ICD-10) and then documented on ED discharge records. I25 and I20 except I20.0 was selected to identify SIHD cases as a previous study reported^23^.

### Air pollution and meteorological data

Daily average concentrations for particulate matter (PM)_2.5_ (fine particulate matter with aerodynamic diameter ≤ 2.5 μm), PM_10_ (fine particulate matter with aerodynamic diameter ≤ 10 μm), sulfur dioxide (SO_2_), nitrogen dioxide (NO_2_), carbon monoxide (CO), and 8-h maximum levels of O_3_ (O_3_-8 h) from January 1, 2013, to December 31, 2020, were obtained from the air quality sharing platform of China. This platform is administered by China’s Ministry of Environmental Protection and displays real-time concentrations of criteria air pollutants in all national air quality monitoring sites around China^24,25^. We utilized the average air pollutant concentrations obtained from all the available air pollution monitoring stations across Shanghai city as a proxy for measuring air pollution exposure. Daily meteorological data, such as daily mean temperature (°C) and relative humidity (%), were collected from the National Weather Data Sharing System of China (http://data.cma.cn)^24^.

### Statistical analysis

We conducted a comprehensive analysis to investigate the links between short-term exposure to various air pollutants and the frequency of ED visits for SIHD^24^. Considering that the daily ED visits exhibited a quasi-Poisson distribution, we employed an overdispersed generalized additive model (GAM) to analyze the data. The model included the average temperature for the current day (the degree of freedom, df = 6), relative humidity for the current day (df = 3), calendar time (df = 8/year), and adjustment for day of the week (DOW) and public holiday (PH)^24,26^. The main model is described as follow:

$${\text{log}}\left( {E\left( {Yt} \right)} \right) = \beta *Zt + ns\left( {time,df = {8}/year} \right) + ns\left( {temperature,df = {6}} \right) + ns\left( {relative\,\,humidity,df = {3}} \right) \, + factor(DOW) + factor\left( {PH} \right) + \alpha ,$$

where *E(Yt)* represents the expected ED visits for CHD on day t, *Zt* refers to various air pollutants concentration on day t, time refers to calendar time which was used to control unmeasured long-term trend, temperature and relative humidity refers to average temperature and relative humidity of the current day, respectively, DOW and PH are dummy variables, *β* is the coefficient for *Zt,* ns refers to a natural cubic smooth function, df refers to the degree of freedom, and α is the intercept. We can calculate the Relative Risk by taking logarithm of *β*.

The main model investigated both single-day lags from the current day (lag0) to five days before (lag5), as well as moving average exposure from the current day to the previous 1 to 3 days (lag01 to lag03). The optimal lag periods were determined based on the maximum estimates and minimum values of *p*^27^. The estimates were reported as excess risk (ER) with a corresponding 95% confidence interval (95% CI). These values represent the percentage change in morbidity associated with a 10-unit increase in different air pollutants^28^.

Afterwards, we excluded the extremely concentrations of air pollutants and then the association between different concentrations of air pollutants and the number of ED visits for SIHD was illustrated using an exposure-response curve. This curve was plotted using a methodology previously reported^24,29^. In the sensitivity analyses, we utilized the raw data to reconstruct the curve.

Subgroup analyses were performed by categorizing the ED visits according to age groups (<50, 50–59, 60–69, 70–79, and ≥80 years), gender (male and female) and season (warm season: April to September; cold season: October to March). In the sensitivity analyses, we varied the df for calendar time from 4 to 16, for average temperature from 4 to 8, and for relative humidity from 3 to 5. Additionally, we conducted two-pollutant models by incorporating air pollutants that had a Spearman's correlation coefficient below 0.7 (Tables S1–S6) in the main model separately. To control a longer time period of ambient temperature, we used the moving average temperature of the current day to the three previous days (temp03), the seven previous days (temp07), and the fourteen previous days (temp014) as substitutes for the average temperature of the present day in the analysis^24^.

R software (version 4.2.3) was used for all analyses, and an association was considered statistically significant when two-sided *p* < 0.05.

### Ethical approval

This study was approved by the Ethics Committee of Shanghai University, all procedures performed in studies involving human participants were in accordance with the ethical standards of the institutional research/review board (IRB)-IRB Number: ECSHU 2023-018. Study participation only required verbal consent, which was obtained from all subjects who participated in this study and was approved by the ethics committee.

## Results

### Description of the study population

The time-series analysis of ED visits revealed that there were small fluctuations in the number of ED visits for SIHD throughout the study period, with a weak seasonal trend (Fig. S2). A total of 18,241 ED visits for SIHD were included (Table 1). The median number of daily ED visits for SIHD varied across different age groups, genders, and seasons (*P *< 0.001). Patients aged 50 years and older accounted for 95.5% of the visits, with females outnumbering males (11,680 females vs*.* 6561 males). Additionally, more SIHD patients were visited during the cold season (52.1%).

**Table 1:** Demographic characteristics of ED visits for SIHD in Shanghai city, 2013–2020.

	Number of visits (%)	Median (IQR) of daily, ED visits for SIHD	P value*
Gender			<0.001
Male	6561 (36.0)	3 (2–5)	
Female	11680 (64.0)	5 (4–7)	
Age (years)			<0.001
< 50	822 (4.5)	1 (1–2)	
50-59	2337 (12.8)	2 (1–3)	
60–69	4522 (24.8)	3 (2–4)	
70–79	4309 (23.6)	2 (2–3)	
≥ 80	6251 (34.3)	3 (2–4)	
Season			<0.001
Warm	8743 (47.9)	7 (5–9)	
Cold	9498 (52.1)	8 (6–11)	

*A Kruskal-Wallis test was used to test the difference between variables.

The time-series patterns of various air pollutants during the study period are displayed in Fig. S3, while the concentrations of different air pollutants, temperature, and relative humidity are presented in Table 2. Generally, all air pollutants exhibit noticeable seasonal trends. The concentration trends of O_3_-8 h were higher in the warm season, while other air pollutants showed higher concentrations in the cold season. The daily mean concentrations of NO_2_, PM_2.5_, PM_10_, SO_2_, O_3_-8 h and CO were 41.40 μg/m^3^ (SD =19.27; range: 5.24 to 140.08 μg/m^3^), 44.15 μg/m^3^ (SD = 32.33; range: 5.33 to 460.98 μg/ m^3^), 61.77 μg/m^3^ (SD =39.22; range: 6.96 to 487.96 μg/m^3^), 13.12 μg/m^3^ (SD = 9.97; range: 4.00 to 106.90 μg/m^3^), 93.12 μg/ m^3^ (SD = 43.15; range: 6.18 to 280.00 μg/m^3^) and 0.78 mg/ m^3^ (SD = 0.88; range: 0.31 to 19.92 mg/m^3^), respectively. The daily average concentrations of NO_2_, PM_2.5_ and PM_10_ exceeded the concentration limits set by the “WHO Air quality guidelines”^30^. In addition, the average daily temperature was 17.52 °C (SD = 8.66; range: -6.10 to 35.00 °C), and the relative humidity was 73.08% (SD = 12.60%; range: 28.00 to 99.00%).

**Table 2:** Summary statistics of daily air pollution and meteorological factors in Shanghai city from January 1, 2013, to December 31, 2020.

	Mean	SD	Minimum	Q1	Median	Q3	Maximum
NO_2_ (μg/m^3^), n=2889	41.40	19.27	5.24	27.71	37.71	51.81	140.08
PM_2.5_ (μg/m^3^), n=2889	44.15	32.33	5.33	22.13	35.67	55.36	460.98
PM_10_ (μg/m^3^), n=2889	61.77	39.22	6.96	35.54	51.09	75.47	487.96
SO_2_ (μg/m^3^), n=2887	13.12	9.97	4.00	7.39	10.54	14.92	106.90
O_3_-8 h (μg/m^3^), n=2870	93.12	43.15	6.18	62.36	88.13	117.13	280.00
CO (mg/m^3^), n=2860	0.78	0.88	0.31	0.55	0.67	0.86	19.92
Mean temperature (°C), n=2921	17.52	8.66	− 6.10	9.90	18.40	24.50	35.00
Relative humidity (%), n=2921	73.08	12.60	28.00	65.00	74.00	83.00	99.00

Q1 means first quartile, Q3 means third quartile.

### Correlation between air pollution and meteorology

Spearman’s correlations among air pollutants and meteorological factors are presented in Fig. S4 and Table S7. PM_2.5_ was strongly associated with PM_10_ (Spearman correlation coefficient, r = 0.88, *p* < 0.001); NO_2_ exhibited a moderate association with PM_2.5_ (r = 0.62, *p* = 0.004), PM_10_ (r =0.60, *p* = 0.008), SO_2_ (r = 0.44, *p* = 0.021) and mean temperature (r = 0.42, *p* = 0.004). SO_2_ demonstrated a moderate association with PM_2.5_ (r = 0.68, *p* = 0.001), PM_10_ (r =0.66, *p* = 0.002) and mean temperature (r = 0.36, *p* = 0.01). Mean temperature exhibited a weak association with PM_2.5_ (r = 0.29, *p* = 0.011) and PM_10_ (r = 0.24, *p* = 0.034). Meanwhile, the associations between O_3_-8 h, CO, relative humidity, and other air pollutants, as well as daily mean temperature, were found to be insignificant.

### Relationship between air pollutants and SIHD

**Figure 1:**
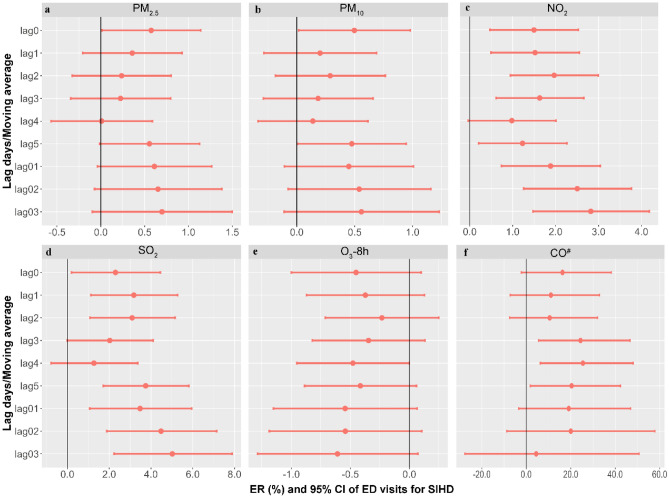
ER (%) and 95% CI of ED visits for SIHD were analyzed in relation to each 10 units increase in various ambient air pollutants. (**a**) PM_2.5_ (μg/m^3^); (**b**) PM_10_ (μg/m^3^); (**c**) NO_2_ (μg/m^3^); (**d**) SO_2_ (μg/m^3^); (**e**) O_3_-8 h (μg/m^3^); (**f**) CO (mg/m^3^).The ER and corresponding 95% CI were derived from an overdispersed generalized additive model, with calendar time, weather conditions, day of the week (DOW) and public holiday (PH) controlled. ^#^indicates that excess risk (%) and 95% CI of ED visits with SIHD were associated with a 10 mg/m^3^ increase in CO concentrations.

The associations between different ambient air pollutants and ED visits for SIHD are presented in Fig. 1. Overall, our findings suggest that there is an elevated risk of ED visits for SIHD with 10 units increase in various ambient air pollutants, such as PM_2.5_, PM_10_, NO_2_, SO_2_, and CO, as well as a decrease in the concentration of O_3_-8 h. We observed significant associations between PM_2.5_ and the number of ED visits for SIHD at lag0, with an ER of 0.58% (95% CI: 0.01%, 1.14%) for every 10 μg/m^3^ increase in PM_2.5_. PM_10_ was also significantly associated with the number of ED visits for SIHD at lag0 and lag5, and the estimates were larger at lag0, with an ER for PM_10_ of 0.50% (95% CI 0.02%, 0.98%) for every 10 μg/m^3^ increase. NO_2_ showed significant positive associations with the number of ED visits for SIHD at lag0 to lag3, lag5, and lag01 to lag03, with the largest effect observed at lag03. The ER for NO_2_ was 2.82% (95% CI 1.47%, 4.18%) for every 10 μg/m^3^ increase at lag03. Similarly, significant positive associations were found between SO2 and SIHD at lag0 to lag2, lag5, and lag01 to lag03, with the largest effect observed at lag03. The ER for SO_2_ was 5.02% (95% CI 2.23%, 7.88%) for every 10 μg/m3 increase. CO was significantly associated with the number of ED visits for SIHD at lag 3 to lag 5, with the largest effect observed at lag 4. The ER for CO was 25.50% (95% CI 6.36%, 48.08%) for each 10 mg/m^3^ increase at lag 4. However, O_3_-8 h showed a significant association with the number of ED visits for SIHD only at lag4, with an ER of -0.48% (95% CI − 0.95%, 0.00%) for every 10 μg/m^3^ decrease in O_3_-8 h. Therefore, in our subsequent analyses, we focused on the effects of PM_2.5_ (lag0), PM_10_ (lag0), NO_2_ (lag03), SO_2_ (lag03), CO (lag4) and O_3_-8 h (lag4).

**Figure 2:**
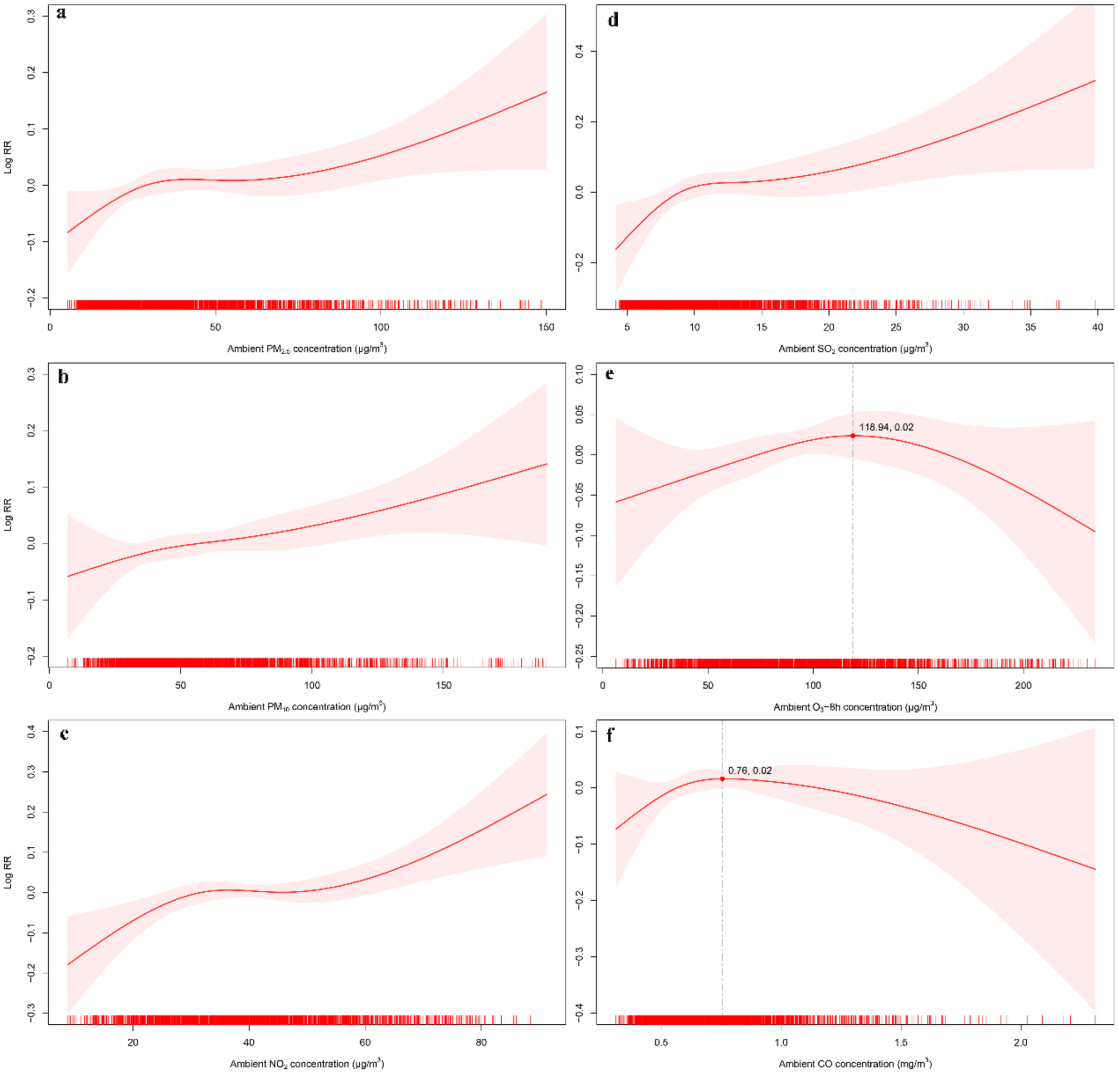
Exposure-response curve for the association between various ambient air pollutants and ED visits for SIHD (Excluding extreme values). The line represents the point estimates, and the shading indicates corresponding 95% CIs, which were derived from an overdispersed generalized additive model, with calendar time, weather conditions, day of the week (DOW) and public holiday (PH) controlled. (**a**) PM_2.5_ (lag0); (**b**) PM_10_ (lag0); (**c**) NO_2_ (lag03); (**d**) SO_2_ (lag03); (**e**) O_3_-8 h (lag4); (**f**) CO (lag4).

Specifically, there were appropriately linear associations between air pollution (PM_2.5_, PM_10_, NO_2_ and SO_2_) and the number of ED visits for SIHD, indicating that higher concentrations of these pollutants corresponded to a greater number of ED visits. No discernible thresholds were observed as the concentrations of these pollutants increased. Regarding O_3_-8 h and CO, we observed a slight initial increase in the number of ED visits for SIHD, reaching a peak at specific air pollution concentrations (O_3_-8 h = 118.94 μg/m^3^, CO = 0.76 mg/m^3^), followed by a subsequent decline (Fig. 2). However, in sensitivity analyses, the dose-exposure plot of PM_10_ and CO exhibits a noticeable change in trend, which may be due to extremely high concentrations of certain air pollutants (Fig. S5).

### Stratified analyses of the association of various ambient air pollutants and SIHD

The results of the stratified analyses are summarized in Table 3. When the data were divided by gender, it was observed that the associations between air pollutants and SIHD were weaker in females than in males. In the age-specific analyses, it was predominantly observed that significant relationships between various air pollutants and SIHD were present in patients aged 60-69 years. In the season-stratified analysis, we observed that the effects of air pollutants on PM_2.5_ and O_3_-8 h remained almost unchanged. The effect estimates were significantly higher during the warm period when analyzing NO_2_, SO_2_ and CO, but the effect was found to be insignificant for CO. In contrast, the effect estimates of PM_10_ were larger during the cold period, but we were unable to detect significant effects during the warm period. For example, the ERs with 95% CIs for the associations of each 10 μg/m^3^ increase in SO_2_ in the warm season and cold season were 14.73% (95% CI 5.44%, 24.84%) and 3.67% (95% CI 0.47%, 6.98%), respectively.

**Table 3:** ER (%) and 95% CI of ED visits with SIHD associated with a 10-unit increase in various air pollution concentrations, stratified by gender, age and season.

	PM_2.5_ (lag0)	PM_10_ (lag0)	NO_2_ (lag03)	SO_2_ (lag03)	O_3_-8 h (lag4)	CO^#^ (lag4)
Total	0.58 (0.01, 1.14)*	0.50 (0.02, 0.98)*	2.82 (1.47, 4.18)***	5.02 (2.23, 7.88)***	− 0.48 (− 0.95, − 0.00)*	25.50 (6.36, 48.08)**
Gender						
Male	0.55 (− 0.19, 1.29)	0.55 (− 0.08, 1.19)	2.61 (0.84, 4.41)**	4.56 (0.91, 8.33)*	− 0.46 (− 1.09, 0.17)	22.82 (− 0.29, 51.28)
Female	0.45 (− 0.18, 1.08)	0.33 (− 0.21, 0.87)	2.40 (0.90, 3.92)**	3.30 (0.22, 6.48)*	− 0.75 (− 1.28, − 0.22)**	20.90 (0.14, 45.97)*
Age group (years)						
< 50	0.18 (− 0.70, 1.07)	0.04 (− 0.73, 0.81)	− 0.17 (− 2.76, 2.49)	1.02 (− 3.96, 6.26)	0.18 (− 0.71, 1.07)	8.79 (− 19.37, 46.78)
50−59	0.45 (− 0.34, 1.24)	0.13 (− 0.56, 0.82)	0.69 (− 1.54, 2.97)	3.73 (− 0.20, 7.82)	− 0.15 (− 0.88, 0.58)	0.24 (− 19.04, 24.10)
60−69	0.69 (− 0.09, 1.47)	0.71 (0.04, 1.38)*	2.20 (0.31, 4.12)*	6.19 (2.01, 10.54)**	− 0.19 (− 0.85, 0.48)	18.84 (− 8.11, 53.69)
70−79	0.16 (− 0.66, 0.99)	0.33 (− 0.37, 1.04)	2.43 (0.49, 4.42)*	2.72 (− 1.37, 6.98)	− 0.28 (− 0.94, 0.39)	24.72 (− 0.95, 57.04)
> 80	0.29 (− 0.46, 1.04)	0.35 (− 0.28, 0.99)	1.50 (− 0.25, 3.27)	2.03 (− 1.62, 5.83)	− 0.39 (− 1.02, 0.24)	13.20 (− 11.25, 44.39)
Season						
Warm	0.57 (− 0.68, 1.83)	0.07 (− 0.93, 1.08)	5.01 (2.01, 8.11)**	14.73 (5.44, 24.84)**	− 0.27 (− 0.86, 0.33)	265.68 (− 15.64, 1485.09)
Cold	0.62 (− 0.01, 1.26)	0.64 (0.09, 1.19)*	1.89 (0.39, 3.41)*	3.67 (0.47, 6.98)*	− 0.33 (− 1.19, 0.54)	25.44 (5.90, 48.59)**

^#^indicates that the units for CO concentration were 10 mg/m^3^, while for other pollutants, the units were 10 μg/m^3^. Associations of statistical significance are listed in the table; ***, ** and * indicate p<0.001, p<0.01 and p<0.05, respectively.

### Sensitivity analyses

Tables S1–S6 show robust results in the two-pollutant models of various air pollutants. Generally, the ER for associations between air pollution (PM_2.5_ and PM_10_) and the number of ED visits for SIHD increased and remained significant only when O_3_-8 h was added in the mode. The ER for associations between air pollution (NO_2_, SO_2_ and CO) and the number of ED visits for SIHD remained significant even after adjusting for other pollutants. However, the results became insignificant for O_3_-8 h. For example, the estimates for the ERs of each 10 μg/m3 increase in NO_2_ slightly decreased to 2.73% (95% CI 1.20%, 4.29%), 2.77% (95% CI 1.22%, 4.34%) and 2.61% (95% CI 1.21%, 4.03%) when PM_2.5_, PM_10_ and SO_2_ were adjusted, respectively. However, they increased to 2.99% (95% CI 1.63%, 4.36%) and 3.02% (95% CI 1.66%, 4.41%) when O_3_-8 h and CO were adjusted, respectively.

When the df of calendar time (from 4 to 16) and temperature (from 4 to 8) were changed, the estimates showed slight variations (Tables S8, 9). For NO_2_, SO_2_ and CO, the results remained robust when the relative humidity (from 3 to 5) was changed or when a longer time period of ambient temperature was controlled (Tables S10, S11). However, because the raw values were already close to the critical value, the results became insignificant for PM_2.5_, PM_10_ and O_3_-8 h (Tables S10, S11).

## Discussion

To the best of our knowledge, this is the first study to simultaneously explore the associations of six types of air pollution (PM_2.5_, PM_10_, NO_2_, SO_2_, CO and O_3_-8 h) with SIHD. Our results revealed significant variations in these associations depending on the type of air pollutant, with the strongest effects observed for CO, followed by SO_2_ and NO_2_. PM_2.5_, PM_10_ and O_3_-8 h showed weak associations with SIHD. Furthermore, the association between various air pollutants and the morbidity of acute SIHD exhibited variations depending on gender, age, and season. Taken together, our results may contribute to a better understanding of the short-term effects of various types of air pollution on acute SIHD.

Few previous studies have explored the relationships between various types of air pollution and ED visits for SIHD^15–20^. We found that the association between air pollution and SIHD varied greatly depending on the specific type of air pollution. For instance, a time-series study conducted in Lima, Peru revealed a significant association between elevated PM_2.5_ levels and an increase in the number of ED visits for IHD at lag0^20^. Similarly, a study in Shanghai, China, which included five clinical subtypes of CHD, reported that a 10 μg/m^3^ increase in present-day PM_10_ concentrations was associated with a respective increase of 0.80% (95% CI 0.22%, 1.37%) in ED visits for ischemic cardiomyopathy^18^. However, other studies have shown a positive correlation between PM_10_ concentration and the number of ED visits for IHD, but the effect was not statistically significant^15,19^. Our study supported the positive association between particulate air pollutants (PM_2.5_, PM_10_) and the number of ED visits for SIHD. Particulate air pollution has been associated with adverse cardiac effects through various biological mechanisms, including enhanced coagulation/thrombosis, a propensity for arrhythmias, acute arterial vasoconstriction, systemic inflammatory responses, and the chronic promotion of atherosclerosis^31^.

In terms of NO_2_, a previous study conducted in Montreal, Canada reported that it contributes to an increased number of ED visits for IHD, with an ER of 5.9% (95% CI 2.1–9.9)^16^. In contrast, a study by Juan Xie et al.^18^ found that there was no significant association between NO_2_ exposure and the number of ED visits for IHD. Our study provided evidence supporting a positive correlation between NO_2_ levels and the number of ED visits for SIHD. The reason for this difference remains uncertain, but it may be attributed to variations in ambient air quality across different regions.

Previous studies have indicated a potential relationship between IHD mortality and CO exposure^32,33^. The study by M. Szyszkowicz ^16^ showed that higher levels of CO were associated with an increased number of ED visits for IHD, with an ER of 5.4% (95% CI 2.3–8.5). Similarly, two previous studies have also reported a positive association between CO and ED visits for IHD, demonstrating a statistically significant effect^15,17^. In our study, we also demonstrated a significant positive association between CO exposure and the number of ED visits for SIHD. The mechanism may involve CO replacing oxygen in the bloodstream, which could potentially impact cardiovascular disease.

Notably, in previous studies, a significant association was found between SO_2_^15,18^ concentrations and ED visits for IHD. However, the effects were not statistically significant. Our study demonstrated a significant association between elevated SO_2_ levels and the number of ED visits for SIHD. It is imperative to conduct more research to better understand which pathways may be responsible for the effect of SO_2_ on SIHD.

Interestingly, a previous study has found a positive correlation between O_3_^15^ levels and ED visits for IHD with no significant. On the contrary, we found a negative correlation between elevated O_3_-8 h levels and the number of ED visits for SIHD. Except for regional differences, this discrepancy is potentially linked to O_3_-8 h levels well below the air quality standards recommended by the WHO in our study.

The observed association between various air pollutants and cardiovascular events may be partially explained by biological mechanisms. First, we used ED visits, as the outcome primarily reflects acute attacks or exacerbations of SIHD. It is worth noting that SIHD is a leading cause of death in the United States and many other countries^4^. Second, several researchers have investigated the association between PMs and CHD, as well as the underlying mechanisms. They found that exposure to PM_2.5_ can damage vascular endothelial cells (ECs), activate inflammatory responses, lead to disseminated intravascular coagulation (DIC), and induce lipid metabolic disorders^34–36^. Moreover, previous studies have demonstrated that various air pollutants, including PM_2.5_, PM_10_, NO_2_, SO_2_, O_3_, and CO, can contribute to oxidative stress pathways and cardiac outcomes. These outcomes may include endothelial dysfunction, atherosclerosis, procoagulant changes, hypertension, and cardiac dysfunction, either independently or through interpollutant interactions^37^. However, the specific mechanisms underlying the impact of various air pollutants on SIHD are still not fully understood. Further research is necessary to gain a better understanding of the potential pathways through which air pollutants affect SIHD. This knowledge will be valuable in guiding the development of effective public health policies.

Certain populations appear to be particularly susceptible to the detrimental effects of air pollution. Haikerwal, A. et al. study^38^ conducted a study that revealed that older adults (aged ≥65 years) and females have a higher risk of IHD. The results were similar to our study, in which over 95% of patients with SIHD were over 50 years old and more than half of them were female. A review also reported that both females and older individuals appear to be more susceptible to the effects of environmental stressors^39^. Given that atherosclerosis (often asymptomatic) is a common comorbidity in older individuals, exposure to high levels of air pollution may increase the risk of coronary episodes. In addition, females typically have a longer lifespan than males and constitute a majority of the aging population^40^.

Our results indicated that a higher number of ED visits for SIHD occurred during the cold season, aligning with previous studies^32,41,42^. This can be attributed to the potential that cold temperatures to raise both systolic and diastolic blood pressure, leading to increased oxygen consumption by the heart^41^. In addition, the subgroup analysis indicated a more pronounced effect of PM_10_ on SIHD during the cold season, which was in general accordance with previous studies^12,18^. Contrary to previous studies^15,18,43^, our findings indicated that the impact of NO_2_, SO_2_, and CO on SIHD was more pronounced in the warm season. The increased risk of cardiovascular disease can be attributed to a combination of factors, including extreme high or low temperatures^6,7^, variations in air pollution mixtures and levels, and population exposure patterns in different seasons. Further investigations are needed to explore the influence of meteorological factors on the relationship between air pollution and SIHD, as well as the underlying mechanisms involved. Moreover, it is crucial to pay more attention to the high levels of PM_10_ during the cold season and the elevated levels of NO_2_, SO_2_, and CO during the warm season in Shanghai.

As mentioned above, cardiovascular disease, especially CHD, imposes a significant burden on both health and society. A modeling study conducted in Australia highlighted the potential benefits of preventing new cases of CHD. If achieved during the 2020–2029 period, it could save over 8000 years of life and gain 104,000 ‘productivity-adjusted life years’, equivalent to a gain of nearly $14.8 billion in GDP^44^. Our study further emphasized the detrimental effects of various air pollutants (PM_2.5_, PM_10_, SO_2_, NO_2_, and CO) on SIHD, underscoring the importance of implementing preventive measures to mitigate SIHD. Firstly, this finding underscores the importance of controlling air pollution. We discovered a positive correlation between air pollutant concentrations and the number of ED visits for SIHD (Fig. 2). Secondly, based on our findings, we recommend implementing specific measures during periods of high air pollution, such as wearing masks and reducing outdoor activities, especially for individuals with SIHD in severe weather conditions.

Our study has several limitations. First, there is the presence of ecological bias inherent in time-series studies. We relied on average values of air pollution data from fixed air-quality monitors instead of individual exposure measurements. This approach may introduce a bias known as the ecological fallacy in the assessment of air pollution exposure. However, previous studies have consistently shown that this type of measurement error tends to underestimate the true risk and lead to conservative estimates of the association^45,46^. Qiu et al.’s study^47^ has shown that this type of misclassification has minimal impact on the results. Second, our study was conducted at a single center and focused on a localized population, which may limit the generalizability of our findings. To address this limitation, we conducted a series of sensitivity analyses over a longer period to enhance the reliability of our findings.

## Conclusion

Our study suggests that short-term exposure to various air pollutants (NO_2_, PM_2.5_, PM_10_, SO_2_ and CO) may act as triggers for inducing SIHD. When the concentration of O_3_-8 h was lower than the threshold recommended by the WHO, exposure to O_3_-8 h was associated with a slightly decreased risk of SIHD. We observed varying associations between different air pollutants and the number of ED visits for SIHD, with variations based on gender, age, and season. By identifying the specific thresholds for each pollutant and understanding their corresponding health effects, appropriate measures can be implemented to mitigate the risks associated with air pollution and promote better cardiovascular health.

### Supplementary Information

Supplementary Information.

## Data Availability

All data generated or analysed during this study are included in this published article and its supplementary information files. The raw data that supporting the findings of this study are available on GitHub at https://github.com/Zhangzhengrenhe163/SIHD.
