# Supplementary material for: Short-term exposure to various ambient air pollutants and emergency department visits for cause-stable ischemic heart disease: a time-series study in Shanghai, China
Source: Sci Rep. 2023 Oct 9;13:16989. doi: 10.1038/s41598-023-44321-1 (PMC10562371; doi:10.1038/s41598-023-44321-1)
Supplement: Supplementary file 1 — Supplementary Information. [file 41598_2023_44321_MOESM1_ESM.docx]

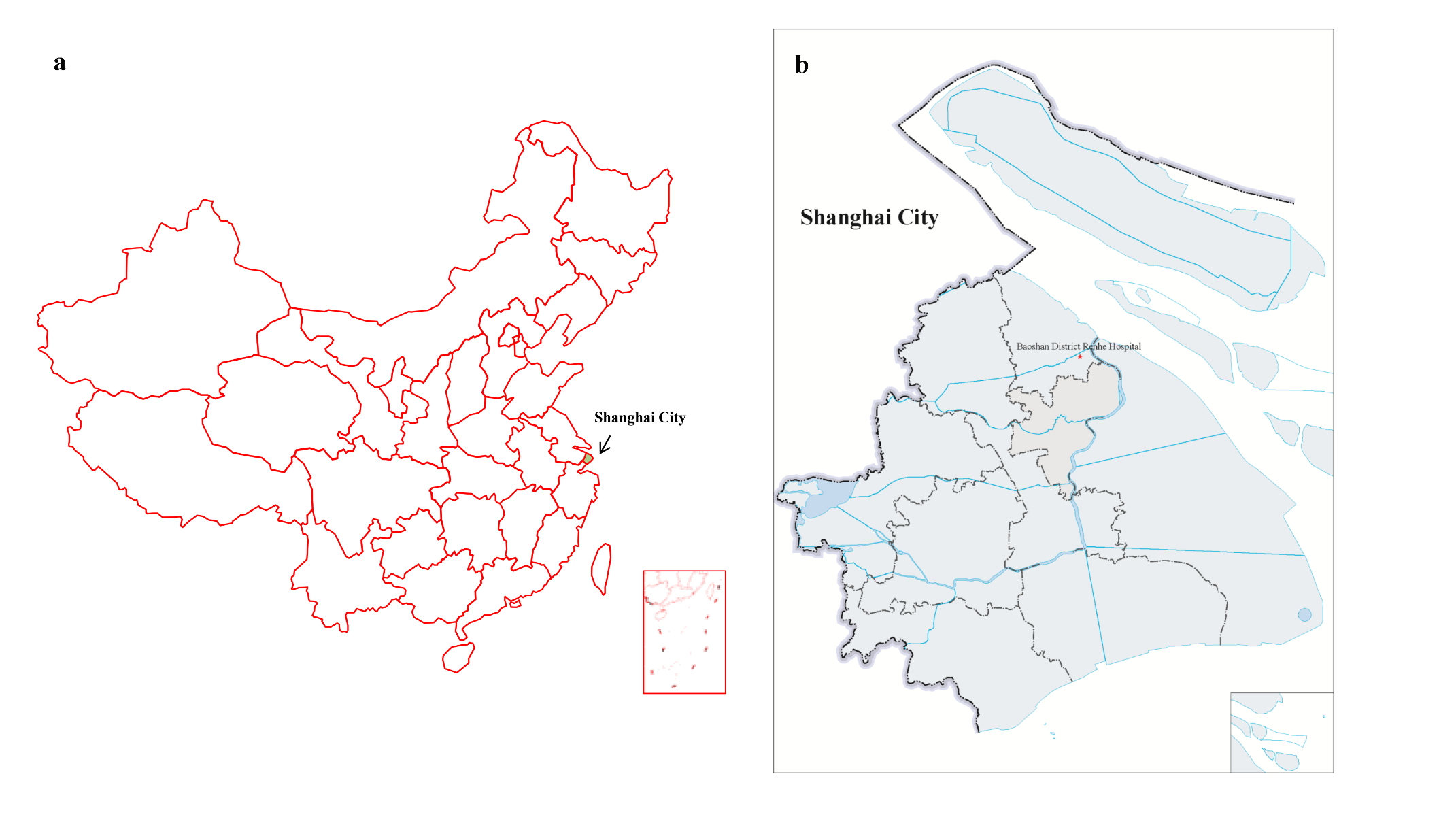


**Figure S1.** The geographical location of Shanghai city, China


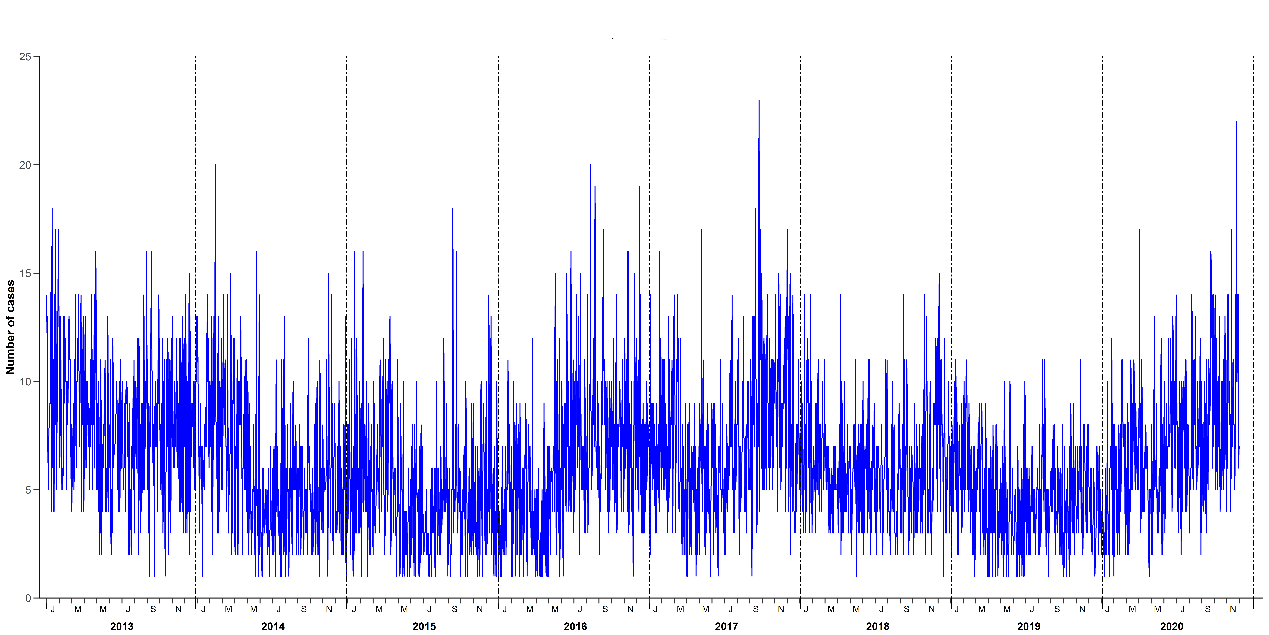


**Figure S2.** Time-series plot of the daily ED visit numbers for SIHD during January 1, 2013 to December 31, 2020.


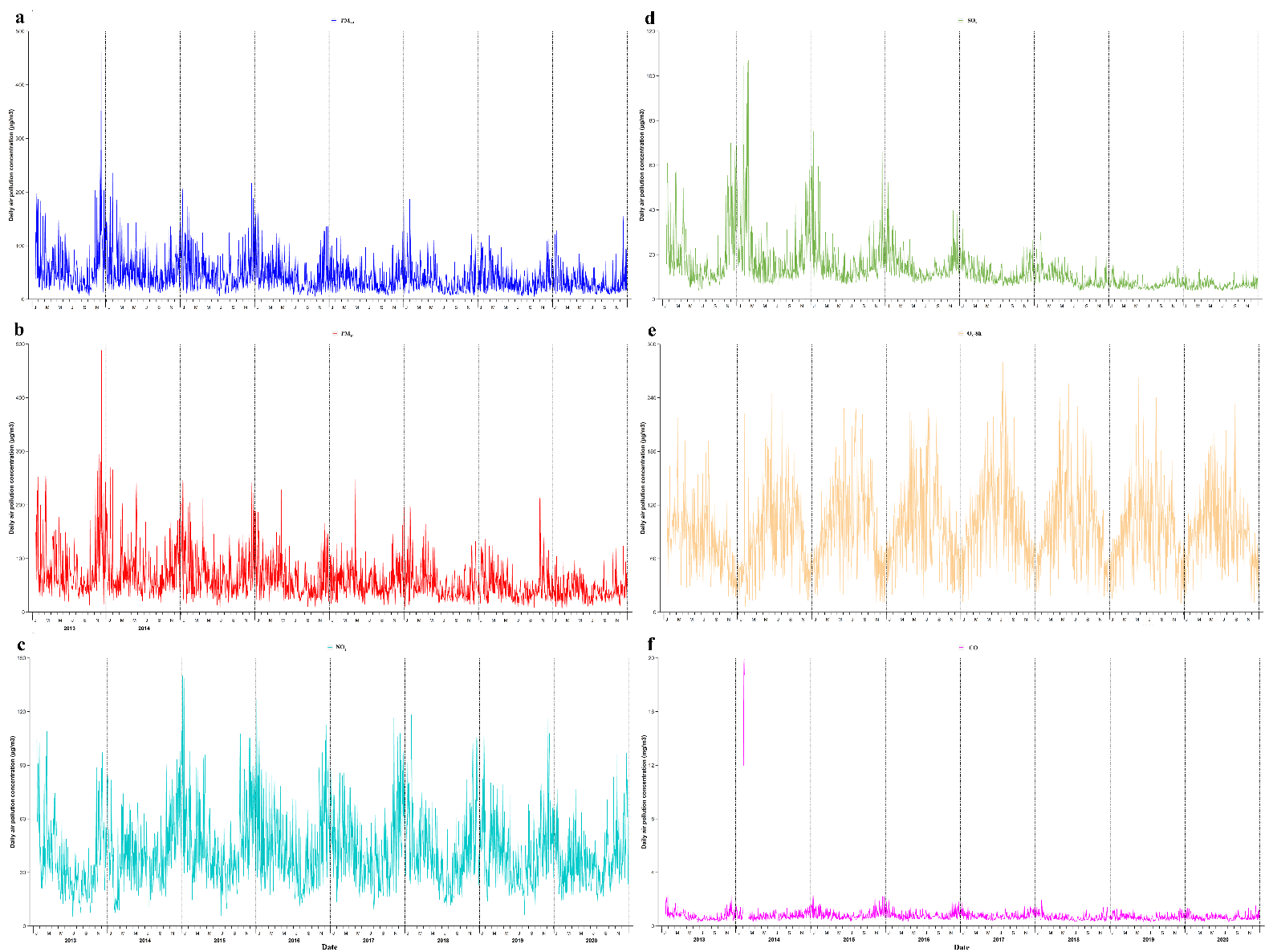


**Figure S3.** Time-series plot of air pollution concentrations during January 1, 2013 to December 31, 2020. a. PM_2.5_; b. PM_10_; c. NO_2_; d. SO_2_; e. O_3_-8 h; f. CO.


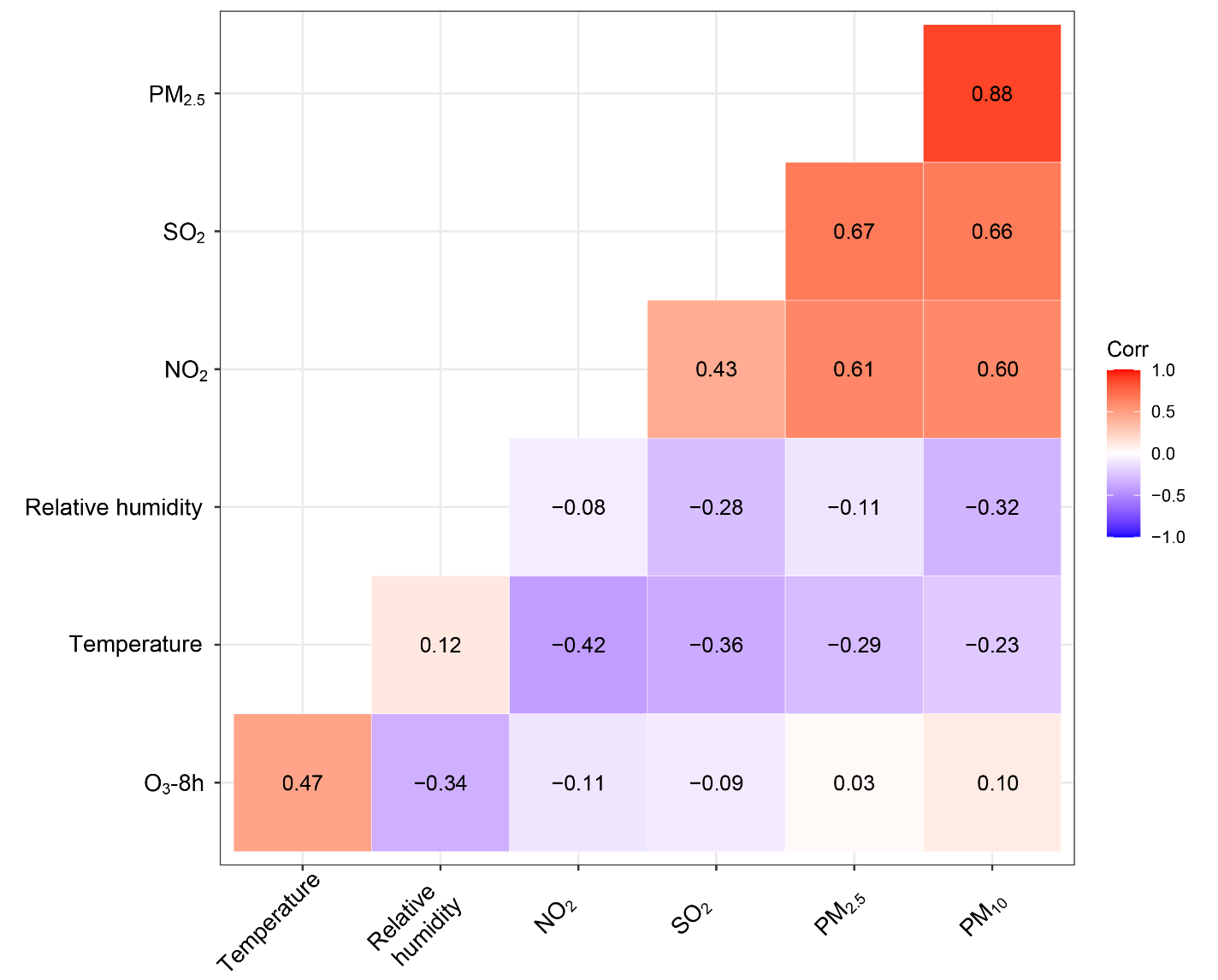


**Figure S4.** Spearman correlation between air pollutants and weather conditions in Shanghai city during the study period.


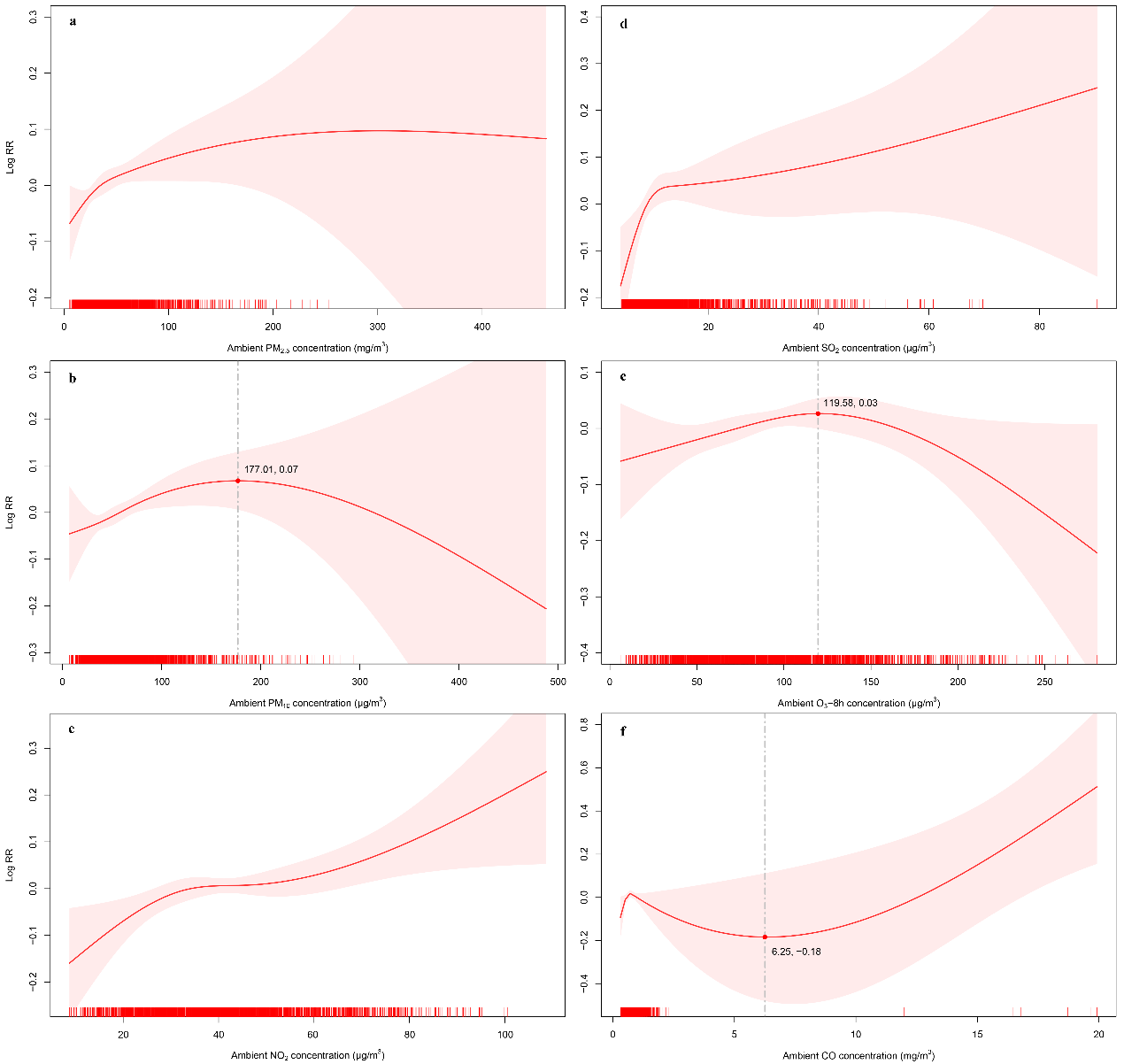


**Figure S5. Exposure-response curve for the association between various ambient air pollutants and ED visits for SIHD (raw data).** The line represents the point estimates, and the shading indicates corresponding 95% CIs, which were derived from an overdispersed generalized additive model, with calendar time, weather conditions, day of the week (DOW) and public holiday (PH) controlled. a. PM_2.5_ (lag0); b. PM_10_ (lag0); c. NO_2_ (lag03); d. SO_2_ (lag03); e. O_3_-8 h (lag4); f. CO (lag4).

**Table S1** Excess risk (%) and 95% CI of ED visits for SIHD in two-pollutant models (PM_2.5_, lag0).

|  | ER % (95% CI) |
| --- | --- |
| Single-pollutant model (PM_2.5_) | **0.58 (0.01, 1.14)*** |
| + NO_2_ | 0.08 (-0.66, 0.82) |
| + SO_2_ | 0.26 (-0.44, 0.97) |
| + O_3_-8h | **0.72 (0.14, 1.30)*** |
| +CO | 0.52 (-0.06, 1.10) |

Note: Associations of statistically significance are in bold, * indicate p<0.05.

**Table S2** Excess risk (%) and 95% CI of ED visits for SIHD in two-pollutant models (PM_10_, lag0).

|  | ER % (95% CI) |
| --- | --- |
| Single-pollutant model (PM_10_) | **0.50 (0.02, 0.98)*** |
| + NO_2_ | 0.07 (-0.55, 0.71) |
| + SO_2_ | 0.33 (-0.24, 0.90) |
| + O_3_-8h | **0.58 (0.09, 1.07)*** |
| +CO | 0.49 (-0.00, 0.98) |

Note: Associations of statistically significance are in bold, * indicate p<0.05.

**Table S3** Excess risk (%) and 95% CI of ED visits for SIHD in two-pollutant models (NO_2_, lag03).

|  | ER % (95% CI) |
| --- | --- |
| Single-pollutant model (NO_2_) | **2.82 (1.47, 4.18)***** |
| + PM_2.5_ | **2.73 (1.20, 4.29)***** |
| + PM_10_ | **2.77 (1.22, 4.34)***** |
| + SO_2_ | **2.61 (1.21, 4.03)***** |
| + O_3_-8h | **2.99 (1.63, 4.36)***** |
| +CO | **3.02 (1.66, 4.41)***** |

Note: Associations of statistically significance are in bold, ***indicate p<0.001.

**Table S4** Excess risk (%) and 95% CI of ED visits for SIHD in two-pollutant models (SO_2_, lag03).

|  | ER % (95% CI) |
| --- | --- |
| Single-pollutant model (SO_2_) | **5.02 (2.23, 7.88)***** |
| + PM_2.5_ | **4.52 (1.50, 7.64)**** |
| + PM_10_ | **4.44 (1.43, 7.54)**** |
| + NO_2_ | **3.94 (1.01, 6.97)**** |
| + O_3_-8h | **5.27 (2.47, 8.16)***** |
| +CO | **5.23 (2.05, 8.51)**** |

Note: Associations of statistically significance are in bold, *** and ** indicate p<0.001 and p<0.01, respectively.

**Table S5** Excess risk (%) and 95% CI of ED visits for SIHD in two-pollutant models (O_3_-8h, lag4).

|  | ER % (95% CI) |
| --- | --- |
| Single-pollutant model (O_3_-8h) | **-0.48 (-0.95, -0.00)*** |
| +PM2.5 | -0.43 (-0.90, 0.05) |
| +PM_10_ | -0.43 (-0.90, 0.05) |
| + NO_2_ | -0.37 (-0.85, 0.11) |
| + SO_2_ | -0.43 (-0.91, 0.04) |
| +CO | -0.48 (-0.95, 0.00) |

Note: Associations of statistically significance are in bold, * indicate p<0.05.

**Table S6** Excess risk (%) and 95% CI of ED visits for SIHD in two-pollutant models (CO, lag4).

|  | ER % (95% CI) |
| --- | --- |
| Single-pollutant model (CO) | **25.50 (6.36, 48.08)**** |
| +PM2.5 | **26.54 (7.21, 49.36)**** |
| +PM_10_ | **26.81 (7.44, 49.67)**** |
| + NO_2_ | **28.35 (8.63, 51.64)**** |
| + SO_2_ | **20.77 (2.06, 42.91)*** |
| + O_3_-8h | **25.97 (6.80, 48.58)**** |

Note: Associations of statistically significance are in bold, ** and * indicate p<0.01 and p<0.05, respectively.

**Table S7**. Spearman correlation between air pollutants and weather conditions in Shanghai city, 2013-2020.

|  | PM_2.5_ | PM_10_ | SO_2_ | NO_2_ | O_3_ | CO | Mean temperature | Relative humidity |
| --- | --- | --- | --- | --- | --- | --- | --- | --- |
| PM_2.5_ | 1 | - | - | - | - | - | - | - |
| PM_10_ | **0.88** | 1 | - | - | - | - | - | - |
| SO_2_ | **0.67** | **0.66** | 1 | - | - | - | - | - |
| NO_2_ | **0.61** | **0.60** | **0.43** | 1 | - | - | - | - |
| O_3_-8h | 0.03 | 0.10 | -0.09 | -0.11 | 1 | - | - | - |
| CO | 0.24 | 0.20 | 0.32 | 0.14 | -0.10 | 1 | - | - |
| Mean temperature | **-0.29** | **-0.23** | **-0.36** | **-0.42** | 0.47 | -0.18 | 1 | - |
| Relative humidity | -0.11 | -0.32 | -0.28 | -0.08 | -0.34 | 0.02 | 0.12 | 1 |

Note: Abbreviations: PM_2.5_ = particulate matter with an aerodynamic diameter smaller than or equal to 2.5 μm; PM_10_ = particulate matter with an aerodynamic diameter smaller than or equal to 10 μm; SO_2_ = sulfur dioxide; NO_2_ = nitrogen dioxide, O_3_ = ozone, CO= carbon monoxide.

Correlations of statistical significance are in bold.

**Table S8** Excess risk (%) and 95%CI of ED visits for SIHD associated with 10 units increase in air pollutions under varying degrees of freedom for the smooth functions of calendar time in single-pollutant models.

| df for time  (per year) | **PM_2.5_ (lag0)** | **PM_10_ (lag0)** | **NO_2_ (lag03)** | **SO_2_ (lag03)** | **O_3_-8h (lag4)** | **CO** ^#^ **(lag4)** |
| --- | --- | --- | --- | --- | --- | --- |
| 4 | 0.42 (-0.19, 1.03) | 0.41 (-0.11, 0.93) | **2.83 (1.42, 4.27)***** | **5.95 (2.97, 9.01)***** | **-0.60 (-1.10, -0.10)*** | **28.48 (7.82, 53.09)**** |
| 5 | **0.60 (0.02, 1.18)*** | **0.58 (0.08, 1.08)*** | **2.48 (1.13, 3.85)***** | **6.37 (3.47, 9.35)***** | **-0.80 (-1.27, -0.32)**** | **32.53 (11.64, 57.32)**** |
| 6 | **0.61 (0.04, 1.18)*** | **0.49 (0.01, 0.98)*** | **2.23 (0.91, 3.58)***** | **5.29 (2.47, 8.19)***** | **-0.71 (-1.18, -0.24)**** | **29.36 (9.37, 53.00)**** |
| 7 | **0.61 (0.05, 1.18)*** | **0.51 (0.03, 1.00)*** | **2.60 (1.27, 3.95)***** | **5.11 (2.32, 7.99)***** | **-0.58 (-1.05, -0.11)*** | **26.63 (7.22, 49.55)**** |
| 8 | **0.58 (0.01, 1.14)*** | **0.50 (0.02, 0.98)*** | **2.82 (1.47, 4.18)***** | **5.02 (2.23, 7.88)***** | **-0.48 (-0.95, -0.00)*** | **25.50 (6.36, 48.08)**** |
| 9 | **0.61 (0.05, 1.18)*** | **0.52 (0.04, 1.01)*** | **2.74 (1.39, 4.11)***** | **5.28 (2.49, 8.16)***** | **-0.50 (-0.98, -0.02)*** | **25.94 (6.70, 48.65)**** |
| 10 | **0.62 (0.06, 1.18)*** | **0.52 (0.04, 1.01)*** | **2.80 (1.45, 4.17)***** | **5.00 (2.20, 7.87)***** | -0.47 (-0.95, 0.01) | **26.21 (6.97, 48.89)**** |
| 11 | **0.60 (0.04, 1.17)*** | **0.53 (0.04, 1.01)*** | **2.86 (1.50, 4.24)***** | **5.19 (2.38, 8.07)***** | -0.48 (-0.96, 0.00) | **25.92 (6.71, 48.59)**** |
| 12 | **0.71 (0.15, 1.27)*** | **0.59 (0.11, 1.07)*** | **2.86 (1.52, 4.23)***** | **5.29 (2.49, 8.16)***** | -0.30 (-0.79, 0.18) | **28.40 (8.98, 51.28)**** |
| 13 | **0.67 (0.11, 1.24)*** | **0.55 (0.07, 1.03)*** | **2.89 (1.54, 4.26)***** | **5.91 (3.09, 8.81)***** | -0.40 (-0.89, 0.09) | **29.91 (10.21, 53.14)**** |
| 14 | **0.70 (0.15, 1.26)*** | **0.57 (0.10, 1.05)*** | **2.66 (1.33, 4.00)***** | **5.65 (2.85, 8.53)***** | -0.27 (-0.76, 0.21) | **27.99 (8.54, 50.93)**** |
| 15 | **0.59 (0.03, 1.15)*** | **0.49 (0.01, 0.97)*** | **3.27 (1.92, 4.63)***** | **4.92 (2.10, 7.82)***** | **-0.54 (-1.02, -0.06)*** | **23.13 (4.44, 45.16)*** |
| 16 | **0.69 (0.14, 1.24)*** | **0.54 (0.07, 1.01)*** | **2.61 (1.30, 3.94)***** | **4.40 (1.53, 7.34)**** | -0.15 (-0.64, 0.33) | **22.28 (3.70, 44.19)*** |

Note: ” ^#^” indicates that the units for CO concentration were 10 mg/m^3^, while for other pollutants, the units were 10 μg/m^3^. Associations of statistically significance are in bold, ***, ** and * indicate p<0.001, p<0.01 and p<0.05, respectively.

**Table S9** Excess risk (%) and 95%CI of ED visits for SIHD associated with 10 units increase in air pollutions under varying degrees of freedom for the smooth functions of temperature in single-pollutant models.

| df for temperature | **PM_2.5_ (lag0)** | **PM_10_ (lag0)** | **NO_2_ (lag03)** | **SO_2_ (lag03)** | **O_3_-8h (lag4)** | **CO** ^#^ **(lag4)** |
| --- | --- | --- | --- | --- | --- | --- |
| 4 | **0.59 (0.03, 1.16)*** | **0.52 (0.04, 1.00)*** | **2.81 (1.47, 4.18)***** | **5.19 (2.40, 8.06)***** | **-0.58 (-1.05, -0.10)*** | **24.45 (5.52, 46.77)**** |
| 5 | **0.57 (0.01, 1.14)*** | **0.51 (0.03, 1.00)*** | **2.88 (1.54, 4.25)***** | **5.02 (2.23, 7.88)***** | **-0.53 (-1.00, -0.05)*** | **26.06 (6.82, 48.76)**** |
| 6 | **0.58 (0.01, 1.14)*** | **0.50 (0.02, 0.98)*** | **2.82 (1.47, 4.18)***** | **5.02 (2.23, 7.88)***** | **-0.48 (-0.95, -0.00)*** | **25.50 (6.36, 48.08)**** |
| 7 | **0.58 (0.02, 1.15)*** | **0.50 (0.01, 0.98)*** | **2.85 (1.51, 4.21)***** | **4.96 (2.18, 7.83)***** | -0.47 (-0.94, 0.01) | **25.70 (6.54, 48.32)**** |
| 8 | **0.59 (0.03, 1.16)*** | **0.51 (0.03, 0.99)*** | **2.85 (1.50, 4.21)***** | **5.02 (2.23, 7.88)***** | -0.48 (-0.95, 0.00) | **25.52 (6.37, 48.12)**** |

Note: ” ^#^” indicates that the units for CO concentration were 10 mg/m^3^, while for other pollutants, the units were 10 μg/m^3^. Associations of statistically significance are in bold, ***, ** and * indicate p<0.001, p<0.01 and p<0.05, respectively.

**Table S10** Excess risk (%) and 95%CI of ED visits for SIHD associated with 10 units increase in NO_2_ (lag03) under varying degrees of freedom for the smooth functions of relative humidity in single-pollutant models.

| df for relative humidity | **PM_2.5_ (lag0)** | **PM_10_ (lag0)** | **NO_2_ (lag03)** | **SO_2_ (lag03)** | **O_3_-8h (lag4)** | **CO** ^#^ **(lag4)** |
| --- | --- | --- | --- | --- | --- | --- |
| 3 | **0.58 (0.01, 1.14)*** | **0.50 (0.02, 0.98)*** | **2.82 (1.47, 4.18)***** | **5.02 (2.23, 7.88)***** | **-0.48 (-0.95, -0.00)*** | **25.50 (6.36, 48.08)**** |
| 4 | 0.52 (-0.05, 1.09) | 0.45 (-0.04, 0.93) | **2.72 (1.38, 4.09)***** | **4.96 (2.17, 7.82)***** | -0.47 (-0.95, 0.00) | **25.38 (6.27, 47.94)**** |
| 5 | 0.53 (-0.04, 1.10) | 0.45 (-0.03, 0.94) | **2.73 (1.39, 4.10)***** | **4.97 (2.17, 7.84)***** | -0.47 (-0.94, 0.01) | **25.22 (6.11, 47.78)**** |

Note: ” ^#^” indicates that the units for CO concentration were 10 mg/m^3^, while for other pollutants, the units were 10 μg/m^3^. Associations of statistically significance are in bold, ***, ** and * indicate p<0.001, p<0.01 and p<0.05, respectively.

**Table S11** Excess risk (%) and 95% CI of ED visits for SIHD associated with 10 units increase in air pollutions when a longer time period of ambient temperature was controlled

|  | **PM_2.5_ (lag0)** | **PM_10_ (lag0)** | **NO_2_ (lag03)** | **SO_2_ (lag03)** | **O_3_-8h (lag2)** | **CO** ^#^ **(lag4)** |
| --- | --- | --- | --- | --- | --- | --- |
| temperature | **0.58 (0.01, 1.14)*** | **0.50 (0.02, 0.98)*** | **2.82 (1.47, 4.18)***** | **5.02 (2.23, 7.88)***** | **-0.48 (-0.95, -0.00)*** | **25.50 (6.36, 48.08)**** |
| temp03 | 0.52 (-0.05, 1.10) | **0.50 (0.01, 0.99)*** | **2.83 (1.46, 4.22)***** | **4.97 (2.11, 7.92)***** | -0.37 (-0.86, 0.11) | **21.51 (2.87, 43.53)*** |
| temp07 | 0.48 (-0.09, 1.06) | 0.48 (-0.01, 0.97) | **2.76 (1.36, 4.17)***** | **4.46 (1.57, 7.43)**** | -0.37 (-0.85, 0.11) | **18.07 (0.10, 39.26)*** |
| temp014 | 0.45 (-0.13, 1.03) | 0.47 (-0.02, 0.96) | **2.76 (1.37, 4.17)***** | **4.11 (1.24, 7.05)**** | -0.36 (-0.83, 0.12) | 16.25 (-1.33, 36.97) |

Note: ” ^#^” indicates that the units for CO concentration were 10 mg/m^3^, while for other pollutants, the units were 10 μg/m^3^. temp03 refers to moving average temperature of the current day to three previous days; temp07 refers to moving average temperature of the current day to seven previous days; temp014 refers to moving average temperature of the current day to fourteen previous days. Associations of statistically significance are in bold, ***, ** and * indicate p<0.001, p<0.01 and p<0.05, respectively.
